# Real-time monitoring of air pathogens in the ICU with biosensors and robots

**DOI:** 10.1186/s13054-022-04222-7

**Published:** 2022-11-14

**Authors:** Linghua Yu

**Affiliations:** Gastroenterology and Hepatology Department, Institute of Liver Diseases, The Affiliated Hospital of Jiaxing University, 1882 Central-South Road, Jiaxing, 314001 Zhejiang Province People’s Republic of China

The management of nosocomial infections in the intensive care unit (ICU) became a tough challenge during the outbreak of COVID-19 [1]. Hospital infection caused by drug-resistant bacteria, viruses, and other microorganisms emerged as a serious problem, especially in the ICU due to its relatively closed space. The airborne pathogenic microorganisms from patients and equipment would spread in the air and contaminate the environment of the ICU [2]. Unfortunately, the prevalence of emerging infectious diseases, such as SARS coronavirus, influenza virus H7N9, and monkeypox, increased the complexity of ICU air microbial communities and further deteriorated this situation [3, 4]. Thus, monitoring airborne pathogens was an essential part of ICU management strategies in preventing hospital-acquired infections. Detecting these airborne pathogens in real-time could help to prevent nosocomial infections and reduce the burden of ICU management in the future.

Several measures were widely adopted in ICU for detecting and monitoring airborne microorganisms. ICU staff used passive collection dishes or active air samplers to collect air samples and then analyze the microbes periodically [5, 6]. But there were obvious limitations to these methods, including cost-effectiveness and inability to achieve real-time detection and warning.

Biosensor technology has been used for decades to test pathogens. Once the target pathogens were detected, the biosensors would change colors or emit fluorescence accordingly [7]. A robot equipped with biosensors could crawl around the ICU and test the airborne pathogens in real-time. The combination of biosensors and robots enabled us to better understand the dynamics of airborne microorganisms and improve infection control measures in the future.

Herein, we introduced a real-time airborne microbes monitoring system to improve the management of nosocomial infection in the ICU (Fig. 1A). The monitoring system was designed as a highly autonomous robot that consists of an automated guided vehicle (AGV), an air sample collector, and an airborne pathogenic microorganisms detecting system (Fig. 1B). Under the control of autopilot and route planning, the robot navigated around the ICU wards and collected air samples. A Mecanum wheel chassis enabled the robot to crawl freely even in narrow spaces. The airborne microbial particles went through the air passage and were kept in the buffer of the sampling tubes. The liquid containing the sampled particles entered the reaction wells through the portal when detection started. The microbes detecting system was designed to have a one-pot reaction procedure, which means the lysis of the pathogenic microorganism, target sequence amplification and detection are all taking place in the same reaction well (Fig. 1C). The target sequences of the pathogenic microorganism were amplified by recombinase polymerase amplification (RPA) and were then detected by a highly sensitive CRISPR-Cas system (CRISPR-12a for detecting DNA sequence and CRISPR-13a for detecting RNA sequence, respectively) [8]. CRISPR-Cas is a rapid, inexpensive, and sensitive nucleic acid detection system with high sensitivity and single base-pair resolution [9]. When aligned with the target sequence, the CRISPR-Cas system will cut the report RNA adjacent to the target sequence and release fluorescence. These biosensors were kept in the reaction wells as freeze-dried. When the fluid containing the sampled microbes enters the wells, the biosensors were rehydrated and the detecting procedure started. Target sequences were released by lysis of the pathogenic microbial particles, amplified by RPA, and were then detected by the CRISPR-Cas system which cuts the reporter RNA and emits fluorescence under the presence of the target sequence (Fig. 1D). The fluorescence event was monitored by a CMOS sensor, and the staff was alarmed about the potential threat of hospital infection through the wireless when the system found the pathogenic microorganisms.

**Fig. 1:**
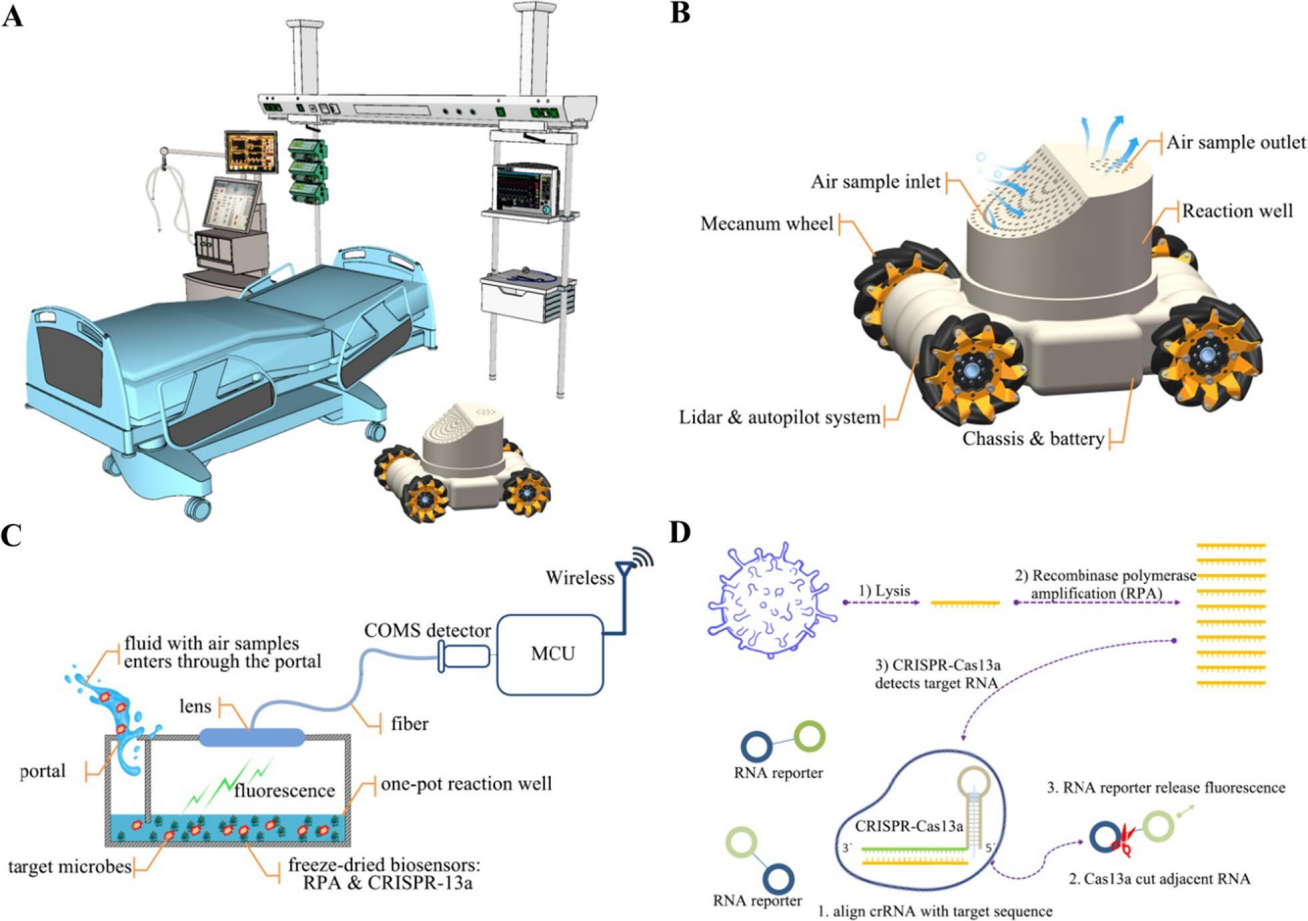
A real-time air microbes monitoring system for nosocomial infection management in ICU. **A.** An automated guided vehicle (AGV) equipped with real-time air microbes monitoring system navigated in the ICU to collect air samples and detect the pathogenic microorganism. **B**. The real-time air microbes monitoring system consisted of an AGV, an air sample collector, and a microbes detecting system. The AGV had a Mecanum wheel chassis, which enable it to navigate freely in narrow spaces. The air sample collector had an air inlet, airway, air outlet, pump, and replaceable sampling tubes. **C.** The airborne pathogenic microorganism detecting system included disposable reaction wells, freeze-dried biosensors (RPA for target sequence amplification, CRISPR-Cas system for high sensitivity detection of the target sequence), fluorescence detecting system (optical lens, fiber, COM detector), and wireless for connecting the Internet of things (IoT). **D.** Target sequences were released by lysis of the pathogenic microorganisms in air samples, amplified by RPA, and were then detected by the CRISPR-Cas system which cuts the reporter RNA and releases fluorescence with the presence of the target sequence

In our preliminary experiments, we verified the real-time airborne microbes monitoring system with *Escherichia coli*. A fragment of the ybbW gene, which was regarded as relatively conservative and specific, was used to identify *Escherichia coli* in our test. RPA primers (forward: 5`-TGCATGATACTGATCGGCAAACTCTGGTCG-3`, reverse: 5`-GCCTTATCGCCGATAACGATAGATGCACGGCTT-3`) and CRISPR RNA (crRNA, 5`-CUUAAUGCAGACAGUUCGGAUCACAUGC-3`) that specific to the ybbW gene were designed with online software. Reagents used in the test mainly included Tris–HCl(pH 7.5), EDTA(pH 9) and Triton X-100 (Sigma Chemical) for lysis reaction, RPA primers and TwistAmp Basic (TwistDx) for RPA reaction, and crRNA, Cas12a (New England Biolabs) and ssDNA fluorescent reporter (Integrated DNA Technologies) for target DNA detection. Our data showed the air sample in ICU wards was positive for *Escherichia coli* and the limit of detection (LoD) was 5 copies per reaction. The result was further confirmed by 16 s rRNA sequencing with air sample collected by an Andersen sampler.

We believed that real-time monitoring of air pathogens in the ICU will provide new insight into the management of nosocomial infections. Combining the biosensors and robot enabled us to monitor the airborne pathogens in real-time, which helped to prevent hospital-acquired infection and ease the burden of ICU management in the future.

## Data Availability

Not applicable.
